# Solo music performance assessment criteria: a systematic review

**DOI:** 10.3389/fpsyg.2024.1467434

**Published:** 2024-10-09

**Authors:** Nádia Moura, Pedro Dias, Lurdes Veríssimo, Patrícia Oliveira-Silva, Sofia Serra

**Affiliations:** ^1^University of Coimbra, CEIS20, Faculty of Letters and Humanities, Coimbra, Portugal; ^2^Universidade Católica Portuguesa, School of Arts, Research Centre for Science and Technology of the Arts, Porto, Portugal; ^3^University of the Azores, Department of Psychology, Azores, Portugal; ^4^Universidade Católica Portuguesa, Research Centre for Human Development, Porto, Portugal; ^5^Universidade Católica Portuguesa, Faculty of Education and Psychology, Porto, Portugal; ^6^Universidade Católica Portuguesa, Faculty of Education and Psychology, Human Neurobehavioral Laboratory, Porto, Portugal; ^7^University of Aveiro, Department of Communication and Arts, Institute of Ethnomusicology – Center for Studies in Music and Dance, Aveiro, Portugal

**Keywords:** instrumental and vocal performance evaluation, judges, rating scales, music pedagogy, music competitions, western classical music, systematic review

## Abstract

Assessment is a crucial aspect of music performance. In pedagogical contexts, an effective assessment process can measure student achievement and inform instructional decisions that contribute to improving teaching and learning. However, music performance assessment is particularly challenging due to its inherent subjectivity, involving personal expression and interpretation, which can lead to divergent opinions. In this PRISMA systematic review (registration DOI: 10.17605/OSF.IO/CSM8Q), we aimed to delimit and analyze solo music performance assessment systems found in the literature to date, including their corresponding evaluation categories and descriptive criteria, rating methodology, and target audience. A search in three main scientific databases (Web of Science, Scopus, ERIC) was conducted using keywords associated with the topic of assessment in the field of solo music performance. Ultimately, 20 papers were selected and examined, resulting in 26 original assessment systems for analysis. Regarding sample characteristics, we found that studies mainly focused on evaluating high school and university students, with music teachers and faculty members serving as primary evaluators. Many assessment systems were designed to be applicable across various instruments, although some were tailored to specific instruments (e.g., piano, voice) and families (e.g., brass, woodwind). Systems typically structured evaluation around technical, interpretative/expressive, and various musical feature categories (e.g., pitch, rhythm, intonation), further elaborated with descriptive items. While five-point Likert scales were commonly used, recent studies indicated a shift towards rubrics for detailed feedback, which aids examiners’ understanding and supports student progress. No differentiation was found in assessment criteria based on students’ learning stages, suggesting an area for improvement in refining these assessment methods. This study identifies gaps and proposes improvements in existing assessment systems, providing a foundation for educators and policymakers to enhance curriculum design and instructional practices in music education.

## 1 Introduction

Assessment is an integral dimension of music performance, both in educational and professional contexts. The assessment process is defined by Payne et al. (2019, p. 43) as “the collection, analysis, interpretation, and applied response to information about student performance or program effectiveness in order to make educational decisions resulting in continual improvement.” Therefore, achieving effective assessments is of extreme relevance, as they do not only provide an overview of the student’s progress in comparison to the expected skills and knowledge projected for a given outcome or learning level, enabling students and teachers to reorganize practices but also reveal areas upon which curricular adaptations can be implemented (Mustul and Aksoy, 2023; Payne et al., 2019; Tabuena et al., 2021).

However, developing reliable systems for music performance assessment presents multiple challenges. First, each musical instrument requires specific skills (e.g., string instrumentalists develop bowing technique, and wind instrumentalists develop breathing technique), demanding that assessment tasks be tailored to each instrument’s unique requirements (Russell, 2014). Second, although ensuring jury expertise, human-based performance evaluation models carry high degrees of subjectivity, often due to poor descriptions (Giraldo et al., 2019; Thompson and Williamon, 2003; Wesolowski et al., 2016). Third, many instrumental and vocal assessment systems put emphasis on pitch and tempo accuracy, neglecting other important dimensions such as interpretation and sound quality (Giraldo et al., 2019). Ultimately, performance-oriented education receives less attention that general classroom music education, resulting in limited research in this area. Considering the identified challenges, it is crucial that systematic reviews provide a framework for addressing these issues.

In a preliminary database search, four narrative reviews were found about the topic of music performance assessment: three articles (Lerch et al., 2020; McPherson and Thompson, 1998; Zdzinski, 1991) and one book chapter (Russell, 2014). In such reviews, multiple assessment systems were identified, including generalized systems applicable to all instruments (Mills, 1991; Russell, 2010b, 2015; Stanley et al., 2002; Thompson and Williamon, 2003; Wesolowski, 2012, 2021; Winter, 1993) and instrument-specific systems (Abeles, 1973; Bergee, 2003; Wrigley, 2005; Wrigley and Emmerson, 2013). Russell (2014) highlighted the role of four nuclear evaluation categories, common to most studies, which significantly predict evaluators’ assessment accuracy: tone and intonation, articulation, rhythmic accuracy, and interpretation or musical effect. There are other studies, however, presenting a dicotomical distinction between categories related to instrumental and vocal technical skills (e.g., accuracy of notes, of rhythm) and interpretation (e.g., dynamics, suitable sense of styles, sense of performance, bodily communication) (Davidson and Coimbra, 2001; Mills, 1991; Stanley et al., 2002). In fact, a subsequent study demonstrated that both technique and musical expression contributed to increases in assessments of overall performance quality, with technique alone also contributing to rating increases in musical expression (Russell, 2015). Nonetheless, as stated by Lerch et al. (2020), the selection of evaluation parameters is highly dependent on the proficiency level of the students and can also vary depending on the culture and musical style of the music being performed. The reviews also called attention to the wide range of rating scales was implemented across studies (McPherson and Thompson, 1998; Zdzinski, 1991), including qualitative (e.g., in Russell, 2010a, strongly agree/agree/disagree/strongly disagree) and quantitative classifications (e.g., in Thompson and Williamon, 2003, ratings from 1 to 10), as well as a variety of assessment levels (e.g., Mills, 1991, uses four levels, while Wrigley and Emmerson, 2013, use seven levels). Earlier reviews advocated for the need to increase reliability and validity of assessment procedures, highlighting the promising results of more systematic approaches, such as the facet-factorial (Zdzinski, 1991) and the importance of considering the influence of personal, cultural, and social biases on the jury (McPherson and Thompson, 1998). These considerations inspired follow-up research related to judge reliability (Bergee, 2003; Hewitt and Smith, 2004; Smith, 2004, to name a few). The more recent review by Lerch et al. (2020), focused on computerized music analysis, presented an overview of the tools and methods which can be used to automatically assess performance parameters not only for evaluation purposes but also for analysis, modelling, and software development. The authors underscore the relevance of developing accessible and reliable automated systems to improve objectivity in performance assessment, a quest that has been long mentioned (McPherson and Thompson, 1998; Zdzinski, 1991). Russell (2014) also corroborated the potential of technology in music assessment, if its equal availability is ensured for all students.

Hence, the absence of a systematic literature review in solo music performance assessment, coupled with the diverse array of instruments, methods, and rating scales identified in this preliminary research, reinforces the need to delimit and characterize evaluation procedures. This systematic review aims to provide a systematized overview of valuable evidence for academics and educators in this field. It builds on previous studies by critically examining generalized and instrument-specific systems, aiming to integrate their strengths while addressing their limitations. Specifically, its goal is to critically analyze solo music performance assessment systems found in the literature to date, including their corresponding evaluation categories, descriptive criteria, rating methodology, and target audience. We intend to establish a generally accepted set of standards and criteria to measure solo performance quality, if possible, adjusted for different musician populations (e.g., basic and advanced learning levels). The main research question driving this study is “What solo music performance assessment systems are reported and implemented in the literature, and how are they characterized?.” This is followed by the specific questions: “What are the main categories of assessment, and which are given the most importance?,” “Within each category, what descriptive items/criteria are provided to the evaluators?,” “What rating methods are adopted (e.g., qualitative or quantitative, type and size of scales)?,” and “How do assessment systems differ between the types of population being evaluated (e.g., children, professionals)?”

## 2 Methods

This systematic review followed the PRISMA updated guidelines (Preferred Reporting Items for Systematic Reviews and Meta-Analyses, Page et al., 2021). Registration in the OSF (Open Science Framework) was also performed (Registration DOI: 10.17605/OSF.IO/CSM8Q).

### 2.1 Eligibility criteria

The current systematic review covered studies that developed and/or implemented music performance assessment systems, analyzing their methodological design (categories/items for assessment, criteria, and rating scales). Given the qualitative nature of our research question, we used the PEO framework: Population – music performers and students, including children, adolescents, higher education students and professionals (no limitations were imposed due to the scarce existing research); Exposure – the process of performance assessment was considered as the exposure; Outcome – assessment systems and corresponding categories, items, criteria, and rating scales.

Inclusion criteria were established to focus on peer-reviewed articles and reviews that provide detailed descriptions of music performance assessment systems, ensuring the inclusion of rigorous and validated studies. The language criteria was expanded to include articles written in Portuguese, as this is the native language of all authors and there are multiple journals using it as primary language. Exclusion criteria, such as the omission of articles referring to general music education rather than performance assessment, were applied to maintain the specificity and relevance of our review. Based on these considerations, the specific inclusion and exclusion criteria applied in this review are as follows:

Inclusion criteria adopted:

1. Articles with relevant data on the theme of music performance assessment and with descriptions of the assessment systems;
2. Reviews or original research articles published in peer-reviewed journals;
3. Articles written in English or Portuguese;
4. Articles that report evaluations targeted at performers or music students (children, adolescents, higher education students, professionals).

Exclusion criteria adopted:

1. Articles referring to assessment systems of general music education rather than music performance;
2. Articles that were marked as “retracted”;
3. Letters to the editor and grey literature.

### 2.2 Information sources and search strategy

Web of Science (all databases), Scopus, and Education Resources Information Center (ERIC) were the chosen databases for our literature search due to their coverage of peer-reviewed articles in the fields of education, social sciences, and music performance. These databases are recognized for their extensive indexing of high-quality academic journals, ensuring that our review encompasses a wide range of relevant studies. The electronic search was conducted on March 18, 2024, using the expression: (“music* perform*” OR “music play*”) AND [title] (analys* OR assess* OR evaluat* OR rat* OR exam* OR criteri* OR jury OR judge*). The previous keywords were chosen to capture a broad spectrum of terms related to music performance assessment while ensuring specificity to our research focus in instrumental and vocal music performance. Filters were applied to limit the results to research articles and reviews in English and Portuguese.

### 2.3 Data collection, selection, and extraction

Outputs were exported to a reference manager software (Mendeley; © 2024 Elsevier Ltd), and duplicates were removed. The selection process was conducted following three stages. In the screening stage, two researchers independently analyzed titles and abstracts following the eligibility criteria to exclude irrelevant references. When eligibility was ambiguous, the full text of the reference was obtained. In the inclusion stage, the same researchers critically appraised the full texts of the selected references for eligibility, and all relevant references were included in the review. Also, at this point, an examination of the bibliography of each study was performed to identify additional relevant studies complying with the inclusion criteria (backward citation searching). The screening and inclusion stages were replicated for the citation searching. In the case of disagreement over the eligibility of studies, a discussion was carried out between the researchers until a consensus was reached.

Researchers then extracted the data from the included references into a Microsoft Excel sheet. The following information was retrieved: author, year, journal, aim, type of study, sample characteristics (age, learning level, musical instruments, context of implementation), assessment system characteristics (name, categories, items, preponderance of items in the final score, criteria, rating methods), results, and limitations (if applicable). Following, data synthesis was conducted through both qualitative and quantitative methods to provide a comprehensive analysis of the findings, including the presentation of tables and summarizing the studies’ evidence through a qualitative approach.

## 3 Results

### 3.1 Study selection

**Figure 1:**
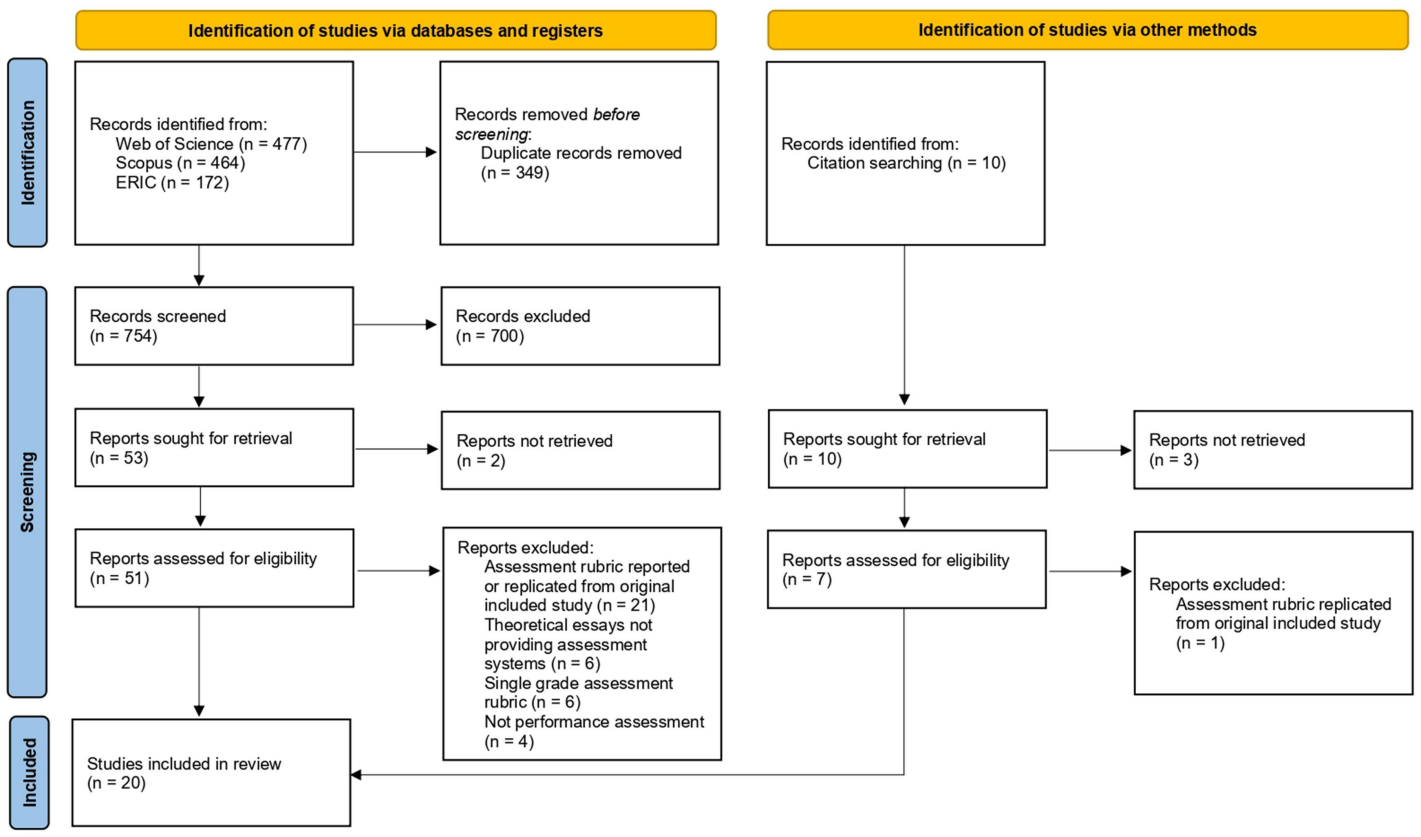
Flow diagram (based on PRISMA statement, Page et al., 2021) summarizing the search procedure.

The selection process is summarized in Figure 1, presenting the PRISMA flow diagram.

A total of 1,113 studies were identified and 754 were retained after the duplicate’s removal. In the screening stage, 700 publications were excluded because they did not fulfil the criteria for inclusion and exclusion, resulting in 53 publications for full-text analysis. Two publications were not fully available online, so publishers and authors were contacted via email, from whom we did not get a response, resulting in a final number of 51 publications. After careful analysis, 37 studies were excluded: 21 studies presented replications or extensions of assessment rubrics originally presented in other included studies (i.e., applying them to ensembles, student self-evaluation, among others), six studies consisted of theoretical articles which, although regarded performance evaluation, did not provide descriptions of assessment systems, six studies implemented single rating assessment systems without specific dimensions or criteria (e.g., single overall rating from 1 to 100), and four studies did not focus on performance assessment (e.g., were focused on listeners’ emotional perceptions).

At this stage, we arrived at 14 publications to include in the review. However, through backward citation searching, we identified 10 additional publications potentially meeting our inclusion criteria. Three of these were impossible to retrieve online and the author informed us that electronic copies of the works were not available. Hence, we analyzed the full text of seven publications. One study was excluded because it presented a replication of the assessment rubric used in another included article, leading us to a total of six publications to add to the review. The systematic review included 20 studies: 18 empirical research articles, one theoretical article, and one narrative literature review. Nineteen studies were written in English and one was written in Portuguese.

Out of the three narrative reviews initially found, two were removed from this systematic review considering that one study (McPherson and Thompson, 1998) reported assessment systems already included under their original empirical research articles, and the other study (Lerch et al., 2020) did not provide information on assessment systems and primarily focused on computer-assisted assessment of sound features. However, we kept one review (Zdzinski, 1991) because it presented additional assessment systems that were deemed relevant to our review.

### 3.2 Sample characteristics

The characteristics of the participants are shown in Table 1.

**Table 1:** Details of included studies—characteristics, samples and methods.

Author (year)	Study type	Study description	Evaluators sample	Evaluated sample	Assessment system
Abeles (1973)	Empirical	Construction and validation of scale	Instrumental music teachers (phase 1, *n* = 50; phase 2, *n* = 32)	Junior high students (*n* = 100) Instruments: clarinet	Clarinet performance rating scale (CPRS)
Fiske (1975)	Empirical	Construction and validation of scale	Teacher-performer specialists (*n* = 14) Instruments: brass, non-brass	High school students (*n* = 32) Instruments: trumpet	Fiske scale
Mills (1987)	Empirical	Construction and validation of scale	Music teachers and music specialist students, and nonspecialists with performance experience (phase 1, *n* = 11; phase 2, *n* = 29)	Students at level of Grade 8 (ABRSM) (phase 1, *n* = 6; phase 2, *n* = 10). Instruments: harp, horn, piano, oboe, violin (phase 1); violin, horn, piano, voice, clarinet, harp, oboe, flute, double bass, trombone (phase 2).	Mills scale
Zdzinski (1991)	Narrative literature review	*Literature review kept to retrieve additional assessment systems not found in empirical studies. For the assessment systems reported in empirical research articles, these were preferred.*			Watkins-Farnum Performance Scale (WFPS) (Watkins and Farnum, 1954; Kidd, 1975*)
Bergee (1993)	Empirical	Replication of author-constructed scale (Bergee, 1987; Bergee, 1988**)	University internal and external faculty members (phases 1, 2, and 3, *n* = 5) Instruments: trumpet, horn, trombone, tuba, percussion	University music students (phase 1, *n* = 10; phases 2 and 3, *n* = 8) Instruments: brass (non-specified)	Brass Performance Rating Scale (BPRS)
Winter (1993)	Empirical	Construction and validation of scale	Qualified musicians and music educators (*n* = 33)	NA (*n* = 1, 3 performances) Instruments: piano	Winter Scale
Saunders and Holahan (1997)	Empirical	Construction and validation of scale	Elementary, secondary, and college-level instrumental music teachers (*n* = 36) Instruments: woodwind and brass	Middle and high school students (Grades 9–12) (*n* = 926) Instruments: woodwind and brass	Woodwind/Brass Solo Evaluation Form (WBSEF)
Davidson and Coimbra (2001)	Empirical	Comparison between quantitative and qualitative assessment	Faculty internal and external highly experienced singers/assessors (*n* = 4) Instruments: voice	2nd year superior-level students (*n* = 21) Instruments: voice	Davidson and Coimbra Scale
Stanley et al. (2002)	Empirical	Interview study and construction of scale	Staff of the Sydney Conservatorium of Music, most with >20 years of performance assessment experience (*n* = 15)	NA *The scales (discussed and created) aimed at superior-level students.*	Sydney Conservatorium Scale Stanley Scale
Zdzinski and Barnes (2002)	Empirical	Construction and validation of scale	Public school string educators, upperclass and graduate string education students, and faculty members (*n* = 50) Instruments: strings	Middle and high school students (*n* = 102) Instruments: strings	String performance rating scale (SPRS)
Bergee (2003)	Empirical	Adaptation of various assessment methods	Faculty members (*n* = 24) Instruments: brass, percussion, woodwind, voice, piano, strings	Undergraduate and graduate music majors and minors (*n* = NA) Instruments: brass, percussion, woodwind, voice, piano, strings	BPRS (Bergee, 1993) Nichols Percussion Scale [Nichols, 1991, as cited in Bergee (2003)] CPRS (Abeles, 1973) Jones Voice Scale (Jones, 1986) Original Piano Scale SPRS (Zdzinski and Barnes, 2002)
Thompson and Williamon (2003)	Empirical	Construction and validation of scale	External professional performing musicians with substantial experience of evaluating at conservatory level (*n* = 3) Instruments: piano, cello, clarinet	Superior-level students (Royal College of Music) (*n* = 61) Instruments: keyboards, woodwind, strings, others (i.e., harp, guitar, brass, voice)	Thompson and Williamon Scale
Barry (2009)	Theoretical	Discussion of politics, issues and successful practices regarding music performance assessment	*Note: Although not empirical, the paper presents a discussion of selected performance evaluation tools and procedures that have been used successfully in music-performance settings.*		Piano Accompaniment and Song Leading Checklist (Benson, 1995, as cited in Barry, 2009) Sample rating scale (adapted from Augustana Percussion Exam) Sample Assessment Rubric for a Preparatory Piano Exam
Ciorba and Smith (2009)	Empirical	Construction and validation of scale	Music faculty members (*n* = 37) Instruments: brass, woodwind, guitar, percussion, piano, strings, voice	Music undergraduate students (*n* = 359) Instruments: brass, woodwind, guitar, percussion, piano, strings, voice	Multidimensional assessment rubric
Russell (2010a)	Empirical	Construction and validation of scale	Public school guitar and string teachers, college guitar professors, undergraduate and graduate music education majors, and professional guitar players (*n* = 67) Instruments: guitar, strings	Professional guitar teachers, college undergraduate and graduate majors, and senior high school freshman, sophomore, junior, and senior (*n* = 55) Instruments: guitar *Total of 100 recordings*	Guitar Performance Rating Scale (GPRS)
Wrigley and Emmerson (2013)	Empirical	Construction and validation of scale	Music faculty members (*n* = 30) Instruments: strings, brass, woodwind, piano, voice	Superior-level student exams (*n* = 829) Instruments: strings, brass, woodwind, piano, voice *(n of students not provided, data collected across semesters can include more than one exam per student)*	Performance examination rating scale (PERS)
Russell (2015)	Empirical	Construction and validation of scale	College undergraduate and graduate music students, university music professors, primary and secondary school music educators, and professional musicians (*n* = 58)	Undergraduate performance and music education majors (*n* = 4) Instruments: French horn, cello, male voice, flute	Aural musical performance quality (AMPQ)
Costa and Barbosa (2015)	Empirical	Construction and validation of scale	Teachers from specialized artistic schools (*n* = 9) Instruments: trumpet	High level 5th musical grade students (*n* = 2) Instruments: trumpet	Scale of evaluation of the musical execution (SEME)
Wesolowski et al. (2017)	Empirical	Construction and validation of scale	Experts experienced in secondary-level instrumental teaching (*n* = 13)	Middle and high school solo and ensemble performances (*n* = 75) Instruments: flute, clarinet, alto saxophone, trumpet, trombone	Music performance rubric for secondary-level instrumental solos (MPR- 2 L-INSTSOLO)
Álvarez-Díaz et al. (2021)	Empirical	Construction and validation of scale	Senior professors of music (*n* = 3) Instruments: piano, viola, clarinet	10 upper secondary students (6^th^ grade of musical studies) Instruments: violin, piano, guitar, percussion, bassoon, flute, tuba	Álvarez-Díaz Scale

*Doctoral thesis unavailable (excluded from analysis). **We were informed by the author that, to his knowledge, these articles, reporting the construction of the scale, are not available in electronic or printed format, hence we included the first available publication mentioning this scale.

Across studies, the number of evaluated participants ranged from one (Winter, 1993) to 926 (Saunders and Holahan, 1997), and the number of evaluator participants ranged from three (Álvarez-Díaz et al., 2021) to 67 (Russell, 2010a). Studies reported performance assessments of students from: junior high only (*n* = 1), middle and high school (*n* = 4), grade 8 ABRSM (Associated Board of the Royal Schools of Music) (*n* = 1), upper secondary music students (*n* = 2), superior-level/university music students (*n* = 7), a combination of professionals, university, and high school students (*n* = 1), or not specified/not applicable (*n* = 4). Performances in the following instruments were included: clarinet only (*n* = 1), trumpet only (*n* = 2), piano only (*n* = 1), voice only (*n* = 1), guitar only (*n* = 1), brass (*n* = 1), woodwind and brass (*n* = 2), strings (*n* = 1), a combination of instruments from varied families and voice (*n* = 7), or not specified/not applicable (*n* = 3).

Evaluator participants were instrumental music teachers (*n* = 4), teacher-performer specialists (*n* = 3), faculty members (*n* = 6), a combination of instrumental teachers, graduate students, and faculty members (*n* = 4), a combination of instrumental teachers and nonspecialists with performative experience (*n* = 1), and not specified/not applicable (*n* = 2). The instrumental expertise of the evaluators was voice only (*n* = 1), guitar only (*n* = 1), trumpet only (*n* = 1), brass (*n* = 1), woodwind and brass (*n* = 1), strings (*n* = 1), mixed panel (varied instrumental families) (*n* = 6), or not specified/not applicable (*n* = 8). Nine studies specifically adopted evaluators with high levels of expertise in the corresponding instrument or instrumental family (e.g., brass) being evaluated.

### 3.3 Assessment systems characteristics

A summary of the 26 assessment systems extracted from the 20 publications analyzed in this review, including names, authors, years of publication, as well as structural characteristics, is presented in Table 2.

**Table 2:** Details of assessment systems retrieved from included studies—categories, items and criteria.

Author (year)	Assessment system	Categories	Items	Rating method
Abeles (1973)	Clarinet performance rating scale (CPRS)	1. Interpretation 2. Intonation 3. Rhythm-continuity 4. Tempo 5. Articulation 6. Tone	1.1. Effective musical communication 1.2. The interpretation was musical 1.3 The piece was played in character 1.4 Played with musical understanding 1.5 Played with traditional interpretation 2.1. Thin tone quality 2.2. Played with a natural tone 2.3 There was a lack of tonal color 2.4 The quality of the tone was rich 2.5 Sounded shallow 3.1. Uneven rhythm 3.2. Smoothness in execution 3.3. Melodic continuation 3.4. Insecure technique 3.5. The rhythm was distorted 4.1. Played out of tune 4.2. Flat in low register 4.3. The intonation was good 4.4. Played overall flat 4.5. Tended to be flat 5.1. Played too fast 5.2. Seemed to drag 5.3. Hurried repeated notes 5.4. Played too slowly 5.5. Rushed 6.1. Squeaked 6.2. Free from tonguing noise 6.3. Attacks and releases were clean 6.4. Tonguing produced thunkie sound 6.5. Accents were played as indicated	5-point scale (Highly disagree, disagree, neutral, agree, highly agree)
Fiske (1975)	Fiske Scale	1. intonation 2. rhythm 3. interpretation 4. technique 5. overall	NA	5-point scale (1–5)
Mills (1987)	Mills scale	1. Nervous 2. Performer did not enjoy playing 3. Performer hardly knew the piece 4. No sense of the piece as a whole 5. Dynamics inappropriate 6. Tempi inappropriate 7. Phrasing inappropriate playing 8. Technical problems distracting 9. Performance hesitant 10. Performance insensitive 11. Performance muddy 12. Performance dull	1. The performer was nervous /confident 2. The performer did not enjoy/did enjoy playing 3. The performer hardly knew/was familiar with the piece 4. The performer did not make sense/made sense of the piece as a whole 5. The performer’s use of dynamics was inappropriate/appropriate 6. The performer’s use of tempi was inappropriate /appropriate 7. The performer’s use of phrasing was inappropriate/appropriate 8. The performer’s technical problems were distracting/ hardly noticeable 9. The performance was hesitant/ fluent 10. The performance was insensitive/sensitive 11. The performance was muddy/clean 12. I found this performance dull/ interesting	4-point bipolar scale, non-specified levels
Zdzinski (1991)	Watkins-Farnum Performance Scale (WFPS)	The Watkins-Farnum Performance Scale consists of a set of 14 exercises (16–36 bars long) of increasing difficulty varying in pitch, rhythm, slurring/articulation, tempo, expression, pause/fermata and repeats. Participants play the exercises in order, and evaluators note each bar in which an error occurs. Per bar, only one error can be scored, hence the possible scores per bar are either one or zero. The maximum possible score on each exercise is a given standard, and the total points scored equals the standard for the exercise minus the number of bars containing an error. Participants continue playing until they score zero on two consecutive exercises. The total score for the test is the cumulative score for all exercises.		12-point score (Satisfatory to Honors)
Bergee (1993) Original unavailable works: Bergee (1987) and Bergee (1988, 1989)	Brass performance rating scale (BPRS)	1. interpretation/musical effect (items 1, 2, 9, 15–19) 2. tone quality/intonation (items 3, 4, 11, 25, 26) 3. technique (items 5–7, 12, 14, 21) 4. rhythm/tempo (items 8, 13, 20, 22–24)	1. Performer plays mechanically 2. Spiritless playing 3. Intonation is inconsistent 4. Plays all registers in tune 5. Performance is clean 6. Poor synchronization of tongue and fingers (slide) 7. Interval leaps are smooth 8. Rhythm flows 9. Superior interpretation 10. Pitch suffers from poor tone production 11. Good intonation at forte volume 12. Lack of clarity in tongued passages 13. Rhythmically accurate 14. Articulation is clean and not percussive 15. Plays rhythms unmusically 16. Ineffective musical communication 17. Neglects style and expression 18. No contrasts in performance 19. Good spirit and drive 20. Tempo not controlled 21. Precise attacks and releases 22. Loud passages rhythmically unsteady 23. Rhythm is unsteady 24. Plays too hurriedly 25. Sound is thin 26. Sound in upper register is pinched and restricted 27. Dynamics are played	5-point scale (Strongly Disagree, Disagree, Neutral, Agree, Strongly Agree)
Winter (1993)	Winter scale	1. Technical 2. Pitch 3. Time 4. Interpretation 5. Overall	1.1. Insecure technique 1.2. Hands well coordinated 1.3. All passages easily executed 1.4. Too heavy handed 1.5. Poor release of notes 1.6. Uneven touch 1.7. Unnecessary finger/hand movement 1.8. Staccato action poor 2.1. Many wrong notes 2.2. Insufficient attention to phrase endings 2.3. Fluent melody lines 2.4. Appropriate balance (melody and chords) 2.5. Fluency impeded by many pauses/stumbles 3.1. Uneven rhythm 3.2. Smooth execution 3.3. Played too fast 3.4. Hurried repeated notes 3.5. Played too slowly 3.6. Accents appropriately performed 3.7. Inconsistent tempo 3.8. Fast passage work needs more control 4.1. Wide dynamic contrasts 4.2. Artistic and skillful execution 4.3. Reflects musical understanding 4.4. Sacrifices style for performance ease 4.5. Sensitive approach to expression 4.6. Haphazard approach to dynamics 5.1. Detailed preparation demonstrated 5.2. Stylistic interpretation 5.3. More hand independence needed 5.4. Played with conviction and sincerity 5.5. Nerves well controlled	6-point scale (HD, D, SD, SA, A, HA) + overall impression (1–10)
Saunders and Holahan (1997)	Woodwind/Brass Solo evaluation form	1. Tone 2. Intonation 3. Technique/Articulation 4. Melodic accuracy 5. Rhythmic accuracy 6. Tempo 7. Interpretation For Scales: 1. Technique 2. Note accuracy 3. Musicianship	Tone (single rating): is full rich, and characteristic of the tone quality of the instrument in all ranges and registers is of a characteristic tone quality in most ranges, but distorts occasionally in some passages exhibits some flaws in production (i.e., a slightly thin or unfocused sound, somewhat forced, breath not always used efficiently, etc.) has several major flaws in basic production (i.e., consistently thin/unfocused sound, forced, breath not used efficiently) is not a tone quality characteristic of the instrument Intonation (single rating): is accurate throughout, in all ranges and registers. is accurate, but student fails to adjust on isolated pitches, yet demonstrates minimal intonation difficulties is mostly accurate, but includes out-of-tune notes. The student does not adjust problem pitches to an acceptable standard of intonation. exhibits a basic sense of intonation, yet has significant problems, student makes no apparent attempt at adjustment of problem pitches. is not accurate. Student’s performance is continuously out of tune Technique/Articulation (Check all applicable): appropriate and accurate tonguing. appropriate slurs as marked. appropriate accents as marked. appropriate ornamentation as marked appropriate length of notes as marked (i.e., legato, staccato) Melodic accuracy (single rating): all pitches/notes accurately. most pitches/notes accurately. many pitches accurately. numerous inaccurate pitches/notes. inaccurate pitches/notes throughout the music, (i.e., missing key signatures, accidentals, etc.) Rhythmic Accuracy (single rating): accurate rhythms throughout. nearly accurate rhythms, but lacks precise interpretation of some rhythm patterns. many rhythmic patterns accurately, but some lack precision (approximation of rhythm patterns used). many rhythmic patterns incorrectly or inconsistently. most rhythmic patterns incorrectly. Tempo (single rating): is accurate and consistent with the printed tempo markings. approaches the printed tempo markings, yet the performed tempo does not detract significantly from the performance. is different from the printed tempo marking(s), resulting in inappropriate tempo(s) for the selection, yet remains consistent. is inconsistent (i.e., rushing, dragging, inaccurate tempo changes). is not accurate or consistent. Interpretation (single rating):	5-point scale (1–5) either continuous (single rating selection) or additive (marked boxes up to 5)
			the highest level of musicality including well-shaped phrases and dynamics. a high level of musicality, but has some phrases or dynamic that are not consistent with the overall level of expression. a moderate level of musicality and musical understanding. only a limited amount of musicality and music understanding. a lack of musical understanding For Scales Technique (Check all applicable): with consistent, even tempo. at required tempo. with appropriate rhythmic pattern. with appropriate articulation as required. evenly, both ascending and descending Note accuracy (Check all applicable): all pitches/notes accurately. most pitches/notes accurately. many pitches/notes accurately. numerous inaccurate notes/pitches. a large number of inaccurate pitches/notes throughout the scale Musicianship (Check all applicable): accurate articulation, clean tonguing. adequate breath control/support. a natural rise and fall of dynamics. adequate and efficient embouchure formation. efficient hand/holding position and commendable erect posture	
Davidson and Coimbra (2001)	Davidson and Coimbra Scale	After the qualitative analysis, the authors were able to conclude that assessors based their evaluations on three main categories: body communication, technical accuracy, and artistry.	1. Free comment 2. Grade 3. Post-performance open questions: 3.1. If the assessor knew the student, and if so, in what capacity; 3.2. How well they knew the pieces being presented; 3.3. Whether the assessors felt that the repertoire was appropriate to the candidate; 3.4. What were the major strengths and weaknesses of the performance; 3.5. What impressions were they left with, and were these impressions different to their initial thoughts.	Free comment + 5-point scale (A – E) + Set of *a posteriori* open questions
Stanley et al. (2002)	1. Sydney Conservatorium Scale 2. Stanley Scale	1.1. Technical 1.2. Musical 2.1. The performance, as a whole, displayed instrumental or vocal control appropriate to the level of examination 2.2. The performance was accurate with respect to rhythm, pitch, articulation, and dynamic 2.3. The performance (where appropriate) was a faithful reading and/or memorisation of the composer’s text 2.4. The performance displayed musically effective production, projection and variation of tone 2.5. The candidate communicated well with other performers, demonstrating good ensemble and listening skills and leadership where appropriate 2.6. The performance communicated an understanding of expressive, stylistic, musical and structural issues 2.7. The performance displayed musical creativity, artistic individuality and effective audience communication	For Vocal Studies: 1.1.1. Technical Facility 1.1.2. Musical Accuracy (Note and Rhythm) 1.1.3. Evenness of Tone 1.1.4. Intonation 1.1.5. Purity of Vowel 1.1.6. Breathing/Posture 1.2.1. Style 1.2.2. Musical Communication 1.2.3. Emotional Impact 1.2.4. Concert Presentation, Flow 1.2.5. Language Facility 1.2.6. Ensemble For Winds: 1.1.1. Intonation 1.1.2. Articulation 1.1.3. Accuracy 1.1.4. Dynamic Contrast 1.1.5. Breathing 1.1.6. Tone Production 1.2.1. Phrasing 1.2.2. Musicianship 1.2.3. Creativity 1.2.4. Emotional Impact For Scale 2., NA	Likert Scale (n of levels not mentioned)
Zdzinski and Barnes (2002)	String performance rating scale (SPRS)	1. Interpretation/musical effect 2. Articulation/tone 3. Intonation 4. Rhythm/tempo 5. Vibrato	1.1. Lack of style in performance 1.2. Very musical 1.3. Melodic phrasing 1.4. Subtle nuances lacking 1.5. Dry-too technical 1.6. Appropriate range of dynamics 2.1. Student is using correct proportion of weight 2.3. Clear articulation produced by left hand 2.4. Maintains proper contact point 2.5. Arm weight draws full sound from string and speed with bow 2.6. Tone is full without harshness on forte 2.7. String crossings are controlled 3.1. Pitch was mostly consistent 3.2. Half steps not close enough 3.3. Consistently good intonation on all strings 3.4. Performer was able to adjust pitch 3.5. Played out of tune 3.6. Minor thirds are sharp 4.1. Uneven rhythm 4.2. Excellent rhythm 4.3. Tempo is not stable 4.4. Rhythm was distorted 4.5. Correct rhythms 4.6. Tempo is steady during technical passages 5.1. Full, rich vibrato 5.2. Vibrato is continuous 5.3. Vibrato is even 5.4. Vibrato is irregular	5-point scale (SA, A, N, D, SD)
Bergee (2003)	1. BPRS (Bergee, 1993) 2. Nichols Scale [Nichols, 1991, as cited in Bergee (2003)] 3. CPRS (Abeles, 1973) 4. Jones Scale (Jones, 1986) 5. Original Piano Scale 6. SPRS (Zdzinski and Barnes, 2002)	1. See Bergee (1993) 2.1. Technique/Rhythm 2.2. Interpretation 2.3. Tone Quality 3. See Abeles (1973) 4.1. Interpretation/Musical Effect 4.2. Tone/Musicianship 4.3. Technique 4.4. Suitability/Ensemble 4.5. Diction 5.1. Interpretation/Musical Effect 5.2. Rhythm/Tempo 5.3. Technique. 6. See Zdzinski and Barnes (2002)	Each category was defined by three items to keep evaluations short, but these are not described in the article. Some scale-specific indications are provided: 2.3. For mallet performance, Tone Quality item “drum tone sounded muffled” was adapted to a more general “tone was characteristic.” 3. No modification outside of the Articulation subscale was required. Under Articulation, the three items dealing with generalized aspects were used (e.g., “free from tonguing noise”) and the two dealing specifically with clarinet articulation (“squeaked”; “tonguing produced thunkie sound”) were ommitted.	5-point scale (SA, A, N, D, SD) + 13-point grade (A+: excellent performance in all respects to F: exceedingly poor performance in all respects)
Thompson and Williamon (2003)	Thompson and Williamon Scale	1. Overall quality 2. Perceived instrumental competence 3. Musicality 4. Communication	1.1. Overall rating of performance quality 2.1. Overall rating of instrumental competence 2.2. Level of technical security 2.3. Rhythmic accuracy 2.4. Tonal quality and spectrum 3.1. Overall rating of musical understanding 3.2. Stylistic accuracy 3.3. Interpretive imagination 3.4. Expressive range 4.1. Overall rating of communicative ability 4.2. Deportment on stage 4.3. Deportment with instrument 4.4. Communication of emotional commitment and conviction 4.5. Ability to cope with the stress of the situation	10-point scale (1–10)
Barry (2009)	1. Piano Accompaniment and Song Leading Checklist (Benson, 1995, as cited in Barry, 2009) 2. Sample rating scale (adapted from Augustana Percussion Exam) 3. Sample Assessment Rubric for a Preparatory Piano Exam	Categories are only provided for Scales 2 and 3: 2.1. Basic Skills: 2.1.1. Tone 2.1.2. Style/Dynamics 2.1.3. Intonation/Sticking 2.1.4. Technique 2.1.5. Grip 2.2. Musicianship: 2.2.1. Phrasing/Dynamic Shaping 2.2.2. Tempo/Pulse 2.2.3. Rhythm 2.2.4. Articulation 3.1. Memorization 3.2. Posture 3.3. Tempo 3.4. Dynamics 3.5. Fingering 3.6. Pedaling	Scale 1: 1. 1. uses correct posture and hand position 1.2. Introduces song 1.3. Cues singers to come in … (counting) 1.4. smiles and looks up when cueing 1.5. plays correct chords 1.6. plays chord changes at correct times 1.7. sings along 1.8. uses proper balance between the hands 1.9. plays in steady tempo throughout 1.10. continues in tempo if chords are missed. Scale 2: 2.1.1. Appropriate mallet/stick choices 2.1.2. Accents (not weight) 2.1.3. Student plays accurately with regard to pitch and intonation. Student chooses appropriate sticking for the selections performed 2.1.4. Student has mastered the relevant technical skills required by the selection(s), 2.1.5. Student establishes and adjusts grip effectively 2.2.1. Student phrases appropriately and intelligently, musical interpretation 2.2.2. Appropriate for the style and character of the work 2.2.3. Rhythms are performed with accuracy and musicality 2.2.4. Student accurately and appropriately conveys score markings. Scale 3 (descriptor per level): 3.1. Not yet: Student needs more than two cues or hesitates more than twice; Almost: Student needs no more than two cues or hesitates no more than twice; Meets Standard: Student needs no cues and hesitates no more than once; Exceeds Standard: Student never needs no cues and never hesitates. 3.2. Not yet: Student sits too close to the keyboard; Almost: Student is at a proper distance from the keyboard but does not have feet flat on the floor; Meets Standard: Student sits at a proper distance from the keyboard with feet flat on the floor; Exceeds Standard: Student sits at a proper distance from the keyboard and maintains a straight but fluid upper body. 3.3. Not yet: Tempo lags or rushes more than twice; Almost: Tempo lags or rushes no more than twice; Meets Standard: Tempo follows the markings in the score and stays with the metronome (set by head judge). Exceeds Standard: Tempo follows score markings and includes liberties taken in the period in which the piece was composed. 3.4. Not yet: Dynamics are incorrect more than twice (i.e., forte is not different than piano). Almost: Dynamics are incorrect no more than twice. Meets Standard: Dynamics follow the markings in the score and are clearly differentiated. Exceeds Standard: Dynamics follow score markings and includes liberties taken in the period in which the piece was composed. 3.5. Not yet: Student misses standard fingering more than once; Almost: Student misses standard fingerings once; Meets Standard: Student follows the score’s fingerings; Exceeds Standard: Student develops more efficient fingering practice. 3.6. Not yet: Student pedals incorrectly two or more times; Almost: Student incorrectly pedals once; Meets Standard: Student follows the score’s pedal markings; Exceeds Standard: Student pedals correctly and artistically.	1. Checklist 2. 5-point scale (0: no evidence, 1: emerging, 2: fair, 3: good, 4: superior). 3. 4-point rubric (Not yet, Almost, Meets Standard, Exceeds Standard).
Ciorba and Smith (2009)	Multidimensional assessment rubric	1. Musical Elements (proficiency with and accuracy of musical elements, including pitch, rhythm, text articulation, and score accuracy) 2. Command of Instrument (represents the student’s ability to control his or her instrument with musical intent) 3. Presentation (presentation demonstrates a lack of sensitivity to musical expression)	Qualitative descriptors (one selection per category): 1. 1 – Accuracy of musical elements does not meet minimal expectations (many noticeable mistakes); 2 – Accuracy of musical elements meets minimal competency (a few noticeable mistakes); 3 – Accuracy of elements is achieved most of the time; 4 – Accuracy of elements is proficient and well established; 5 – Precise demonstration of musical elements is demonstrated. 2. 1 – Command of instrument is below minimum expectations (demonstrates little technical control of instrument); 2 – Command of instrument demonstrates potential for musical growth; 3 – Command of instrument has achieved a point where musical maturity is possible; 4 – Command of instrument is proficient; 5 – Command of instrument demonstrates potential for professional success. 3. 1 – Presentation demonstrates a lack of sensitivity to musical expression; 2 –Presentation demonstrates a potential for musical growth; 3 – Ability to present a musical performance has achieved a point where musical maturity is possible; 4 – Presentation demonstrates that the ability to perform musically is proficient; 5 – Ability to perform musically demonstrates potential for professional success.	5-point rubric (1–5)
Russell (2010a) Original work (thesis): Russell (2010b)	Guitar performance rating scale (GPRS)	interpretation/musical effect technique rhythm/tempo tone intonation	1.1. Melodic expression 1.2. No contrasts in performance 1.3. The interpretation was musical 1.4. Spiritless playing 1.5. Performance not expressive 1.6. Performance reflected sensitivity 1.7. Melodic phrasing 2.1. Tone is strong 2.2. Tone is full 2.3. Thin tone quality 2.4. Tone is rich 2.5. Sound is clear and resonant 2.6. Tone quality is beautiful 2.7. There was a lack of tonal color 3.1. String crossing is controlled and smooth 3.2. Played fluently 3.3. Poor synchronization of pick and frethand fingers 3.4. Lack of clarity in picked passages 3.5. Flubbed 3.6. Attacks were clean 4.1. The tempo was steady 4.2. Correct rhythms 4.3. Off-beats played properly 4.4. Hurried repeated notes 4.5. Lack of a steady pulse 4.7. Tempo not controlled 4.8. The tempo was in good taste 5.1. Played out of tune 5.2. Intonation is good 5.3. Intonation is inconsistent 5.4. Ignored key signature	5-point scale (5 = Strongly Agree, 4 = Agree, 3 = Neutral, 2 = Disagree, 1 = Strongly Disagree)
Wrigley and Emmerson (2013) Other PERS retrieved from author’s doctoral thesis Wrigley (2005)	Performance examination rating scale (PERS)	Piano: 1. Technical mastery and control 2. Sound quality 3. Convincing musical understanding Strings: 1. Technique 2. Musical understanding and performance Brass: 1. Technical preparation 2. Sound production 3. Musical interpretation Woodwind: 1. Technical control 2. Sound production 3. Musicality and interpretation Voice: 1. Technique 2. Interpretation 3. Musicality 4. Communication	Piano: notes: accurate and secure physically: comfortable and at ease tempo: choice well judged and steady tempo control rhythm: accurate and secure or stable control articulation: clear confident: assertive, flair memory: accurate, secure and reliable 2.1. tone, color or dynamics: variety or range and shading or depth 2.2. phrase: sufficient phrasing or shape 2.3. pedal: clear, accurate and refined 2.4. energy: high drive, forward movement, vitality or verve 2.5. flowing: highly fluent or fluid 2.6. texture: clear 2.6. projection: good 3.1. mood or emotion: well conveyed 3.2. ideas, structure, style and character: deeply understood 3.3. musically: very convincing Strings: body: comfortable, at ease or relaxed body, technique, lh, rh playing bow: high level of control and clear articulation tone: full tone, sound quality or color intonation: accurate, secure and/or reliable vibrato: appropriate speed, flexible width and expressive memory: secure and reliable tempo: choice well judged and steady tempo control rhythm: accurate and secure or stable control phrase: well sustained, sensitive and imaginative phrase, line or shape dynamics: high dynamic range/variety or contrast mood/ feeling range: high degree of range or contrast of expression vitality: high energy, drive, buoyancy or vitality free and/or flowing: highly fluent or fluid style and character: deep awareness and understanding ideas: imaginative musical ideas conveyed with conviction ensemble: high degree of balance and collaborative awareness confident performance Brass: notes: accurate and secure rhythm: accurate intonation: accurate dynamics: contrast tempo register/range: upper, lower stamina/endurance memory: accurate 2.1. tone/sound: clear 2.2. airflow/breathing: efficient	7-point scale (generally inadequate throughout; limited throughout; inconsistent throughout; moderately consistent throughout; mostly consistent throughout; consistent command throughout; complete mastery throughout)
			2.3. articulation: clear 2.4. projection: good 3.1. musical/expressive: lyrical, drama, excitement etc. 3.2. style/interpretation 3.3. phrase/shape 3.4. confident 3.5. ensemble: balance, interaction and knowledge woodwind: notes: accurate and secure rhythm: accurate and secure articulation: clear memory: secure 2.1. tone/color: clear, even, register, vibrato 2.2. breath/air: efficient 2.3. reed intonation: accurate, control 3.1. musical/expressive: mood, lyrical, drama, spirit, energy, etc. 3.2. style/character/interpretation: sensitivity 3.3. phrase/shape: musical, legato 3.4. dynamics: contrast 3.5. tempo: steady, secure 3.6. projection 3.7. confidence 3.8. ensemble: balance, interaction and knowledge voice: articulation: clarity, freedom registration: low, high, balanced, tessitura intonation: accuracy tone/color: chiaro scuro, vibrancy, clarity air/breath: appoggio, energy tension: body alignment, ease freedom 2.1. text: accuracy, clarity 2.2. interpretation: expressive, insightful, tone color 2.3. characterization: stage presentation, convincing 2.4. insight: meaningful, imaginative 3.1. style: insightful, musical integrity 3.2. phrase: direction, shape 3.3. dynamics: contrast, choice 3.4. rhythm and tempo: accuracy, choice 4.1. communication: expressive, honest, committed, engaging 4.2. poise/confidence	
Russell (2015)	Aural musical performance quality (AMPQ)	1. tone 2. intonation 3. articulation 4. rhythmic accuracy 5. tempo 6. dynamics 7. timbre 8. interpretation 9. technique 10. musical expression 11. overall quality perception	1.1. Tone is strong 1.2. Tone is full 1.3. Thin tone quality 1.4. Sound is clear 2.1. Played out of tune 2.2. Performer was able to adjust pitch 2.3. Intonation is inconsistent 2.4. Intonation is good 3.1. Correct rhythms 3.2. Off-beats played properly 3.3. Rhythm was distorted 3.4. Insecure rhythm 4.1. Poor synchronization 4.2. Attacks and releases were clean 4.3. Impeccable articulation 4.4. Articulation is overly percussive 5.1. Tempo is steady 5.2. Tempo not controlled 5.3. The tempo was in good taste 5.4. Lack of a steady pulse 6.1. Dynamics are played 6.2. Dynamics used to help phrasing 6.3. Good dynamic contrast 6.4. Appropriate dynamics 7.1. Timbre was harsh or strident 7.2. Demonstrated a singing quality 7.3. Lacked resonance 7.4. Timbre appropriate for style 8.1. The interpretation was musical 8.2. Lack of style in performance 8.3. Effective musical communication 8.4. Melodic phrasing 9.1. Made numerous errors in technique 9.2. Insecure technique 9.3. Precision is lacking 9.4. Played fluently 10.1. Performance not expressive 10.2. Performance reflected sensitivity 10.3. Melodic expression 10.4. Spiritless playing 11.1. Overall quality lacking 11.2. Excellent performance overall 11.3. Poor performance quality 11.4. Quality of performance is good	4-point scale (4 = Strongly Agree, 3 = Agree, 2 = Disagree, 1 = Strongly Disagree)
Costa and Barbosa (2015)	Scale of evaluation of the musical execution (SEME)	1. Materials 1.1. Sensorial (capacity of exploring contact with the trumpet) 1.2. Manipulative (technical manipulation and control) 2. Expression 2.1. Personal (capacity of playing expressively and with musical taste) 2.2. Vernacular (expressive sense according to the established conventions of the musical language) 3. Shape 3.1. Speculative (capacity of controlling expressive details and highlight the piece’s structure) 3.2. Idiomatic (capacity of playing according to technical and aesthetic options according to the musical epoque and style) 4. Value 4.1. Symbolic (refinement of the previous parameters in combination with personal interpretation of the piece) 4.2. Systematic (technical mastery, communication, emotion, and autonomy)	1.1.1. Posture (body/embouchure) 1.1.2. Breathing control 1.1.3. Sound emission 1.2.1. Atack 1.2.2. Articulation 1.2.3. Register/tessitura 1.2.4. Tuning 1.2.5. Sound quality 2.1.1. Respect for the musical text 2.1.2. *Tempo* choice 2.1.3. Rhythmic stability 2.1.4. Use of different dynamic levels 2.2.1. Rhythmical organization of phrases 2.2.2. Melodic organization of phrases 2.2.3. Fluency of musical discourse 2.2.4. Expressivity of musical discourse 3.1.1. Security of musical discourse 3.1.2. Dynamic control and variety 3.1.3. Understanding of musical structure 3.2.1. Notion of musical style 3.2.2. Identification with epoque-related aesthetic options 4.1.1. Refinement of expressive and structural details 4.1.2. Compromise between interpretation and musical style/shape 4.2.1. Excellent technical mastery 4.2.2. Capacity of communicating and conveying emotion 4.2.3. Capacity of self-regulation	4-point scale (insufficient, sufficient, good, excellent).
Wesolowski et al. (2017)	Music Performance Rubric for Secondary-Level Instrumental Solos (MPR-2 L-INSTSOLO)	1. technique 2. tone 3. articulation 4. intonation 5. visual 6. air support 7. melody 8. expressive devices	1.1. Finger/slide dexterity 1.2. Coordination between tongue and fingers/slide 2.1. Tone quality in varying registers 2.2. Tone while executing expressive gestures 3.1. Consistency of articulation 4.1. Intonation accuracy 5.1. Body posture 5.2. Instrument angle 5.3. Head position 5.4. Arm position 5.5. Wrist position 5.6. Hand position 5.7. Embouchure/flexibility 5.8. Cheeks 5.9. Jaw movement 6.1. Breath intake 6.2. Sufficiency of air 6.3. Air support in various registers of the instrument 7.1. Note accuracy 7.2. Communication of musical phrases 7.3. Connection of phrases 7.4. Inflection at cadence points 8.1. Stylistically related dynamics 8.2. Contrast in dynamics 8.3. Subdivision of the rhythm 8.4. Appropriateness of tempo 8.5. Steadiness of pulse 8.6. Expressive pulse and tempo fluctuation	2 to 4-point qualitative rubric, depending on the category To access descriptors of each category, see Appendix A at http://bcrme.press.illinois.edu/media/215/
Álvarez-Díaz et al. (2021)	Álvarez-Díaz Scale	1. Technical Level 2. Quality of interpretation 3. Stylistic coherence 4. Difficulty of the repertoire 5. Stage presence The norms in competition set weightings to the five criteria as follows: Technical level 30%, Performance quality 30%, Stylistic coherence 10%, Difficulty of chosen pieces 20%, Stage presence 10%	Select one level per category: Level 1 (1–2 points): Limited control in the production and continuity of sound and of the intonation. Limited control of mechanical skills. Limited control of the pulse and sense of rhythm. Level 2 (3–4 points): Appropriate control in the production and continuity of sound and of the intonation. Appropriate control of mechanical skills. Appropriate control of the pulse and sense of rhythm. Level 3 (5–6 points): Excellent control in the production and continuity of sound and of the intonation. Excellent control of mechanical skills. Excellent control of the pulse and sense of rhythm. 2.1. Level 1 (1–2 points): The coherence of tempi in the piece and its parts is irregular. The control of phrasing and ornamentation is irregular. Limited control of nuances, sound levels and dynamics. 2.2. Level 2 (3–4 points): The coherence of tempi in the piece and its parts is appropriate. The control of phrasing and ornamentation is appropriate. Appropriate control of nuances, sound levels and dynamics. 2.3. Level 3 (5–6 points): The coherence of tempi in the piece and its parts is excellent. Excellent control of phrasing and ornamentation. Excellent control of nuances, sound levels and dynamics. 3.1. Level 1 (1–2 points): Limited control of the characteristics which identify the artistic trend of a musical period, or genre, or a composer style in the pieces performed and the relationship between its parts. 3.2. Level 2 (3–4 points): Appropriate control of the characteristics which identify the artistic trend of the musical period, or genre, or the composer style in the pieces performed and the relationship between its parts. 3.3. Level 3 (5–6 points): Excellent control of the characteristics which identify the artistic trend of the musical period, or genre, or the composer style in the pieces performed and the relationship between its parts. 4.1. Level 1 (1–2 points): The level of virtuosity proposed is low and/or significant parts of the pieces are excluded. 4.2. Level 2 (3–4 points): The level of virtuosity proposed is adequate and significant parts of the pieces are not excluded. 4.3. Level 3 (5–6 points): The level of virtuosity proposed is extremely high and significant parts of the pieces are not excluded. 5.1. Level 1 (1–2 points): Little naturalness of gesture, poor memorization and/or independence from the score. Little care about staging or self-control throughout the performance. 5.2. Level 2 (3–4 points): Appropriate naturalness of gesture, good memorization and/or independence from the score. Appropriate care of staging and self-control throughout the performance. 5.3. Level 3 (5–6 points): Excellent naturalness of gesture, flawless memorization and/or independence from the score. Excellent care of staging and self-control throughout the performance.	6-point qualitative rubric (final score ranging from 5–30 points)

Most assessment systems were designed for application across instruments (*n* = 11) but there were also family-specific (*n* = 6), and instrument-specific (clarinet, *n* = 1; guitar, *n* = 1; trombone, *n* = 1; percussion, *n* = 2; voice, *n* = 1; piano, *n* = 3) systems.

The first-level assessment categories ranged from two (Benson, 1995, as cited in Barry, 2009; Stanley et al., 2002; Wrigley and Emmerson, 2013) to 12 categories (Mills, 1987) across studies, although most recurrently three, four or five categories were implemented. Technical-related categories were the most frequent (19 studies used the term technique, whereas others defined it as command of instrument, instrumental control, or instrumental competence). Expressive-related categories were also recurrent, emerging under the terms interpretation (14 studies), expression (5), musical understanding (4), musical communication (1), musicality (3), musicianship (3), and artistry (1). Following, we found tone/timbre/sound quality (15), intonation/pitch/melodic accuracy (14), rhythm (13) and tempo (11), articulation (8), dynamics (6), and phrasing (3). While some studies considered rhythm and tempo as separate categories (e.g., Abeles, 1973), others joined them (e.g., Bergee, 1993). Four studies included an additional category related to overall quality. Eight systems further comprised categories related to presentation, confidence, visual, stage presence, and audience communication. Five systems included one category related to the adequacy of the interpretation regarding the musical style and epoque. Two studies included categories of body communication and posture. Moreover, the Álvarez-Díaz Scale (2021) was the only system to consider the difficulty of the repertoire as a category, and the Woodwind/Brass Solo Evaluation Form (Saunders and Holahan, 1997) provided a reduced version for musical scales’ assessment. In instrument- and family-specific systems, idiosyncratic categories were identified, including diction and language facility (for voice), sticking or grip (for percussion), air support, tongue, or vibrato (for winds), memorization, fingering, or pedaling (for piano), and vibrato (for strings).

The most common logic adopted across studies was to select a small set of first-level categories and further expand them into multiple second-level items. However, four studies presented different organizations. Mills’ categories (1987) consisted of 12 statements (e.g., performer hardly knew the piece), which were transposed into 12 bipolar items (e.g., the performer hardly knew/was familiar with the piece). Costa and Barbosa (2015) also presented differing categorical terminologies (materials: sensorial and manipulative, expression: personal and vernacular, shape: speculative and idiomatic, value: symbolic and systematic). Nevertheless, these categories become closer to others in their more objective item form (e.g., tuning, sound quality, notion of musical style). The Watkins-Farnum Performance Scale (1954) also derived significantly from other methodologies, as it consisted of 14 exercises of increasing difficulty in varied musical features (e.g., pitch, rhythm, slurring/articulation, among others) which are played orderly by the evaluated participants. Evaluators score each exercise’s performance by considering the participants’ errors, producing a final score for the test. When participants score zero on two consecutive exercises, they stop the test (see Table 2 for more information). On the other hand, Davidson and Coimbra (2001) arrived at three main *a posteriori* categories (body communication, technical accuracy, artistry) based on evaluators’ ratings and qualitative comments and open-ended responses.

The second-level items ranged from 10 (Stanley et al., 2002) to 44 items (Russell, 2015) across studies, with each of the previous categories commonly being expanded onto multiple items. Three systems did not present items as two comprised a direct rating per category (Fiske, 1975; Stanley et al., 2002) and the other, although mentioning that each category was defined by three items to keep evaluations short, did not provide descriptions in the corresponding article (Bergee, 2003). Items are reported in detail in Table 2.

The rating scales retrieved can be organized into three types: rating scales (*n* = 19), rubrics (*n* = 4), checklists (*n* = 2), and combined checklist and rubric (*n* = 1). Unlike traditional rating scales, rubrics provide detailed information for each score level.

In terms of the number of levels within these scales, the distribution is as follows: 14 systems used 5-point scales (qualitative, *n* = 10; qualitative rubric, *n* = 2; quantitative, *n* = 1; A – E system, *n* = 1), four used 4-point scales (qualitative, *n* = 2; qualitative rubric, *n* = 1; position only, *n* = 1), one adopted a 2 to 4-point qualitative rubric depending on the category; two used 6-point scales (qualitative, *n* = 1; qualitative rubric, *n* = 1), one used a 7-point qualitative scale, one used a 10-point quantitative scale, and one did not provide information. Two studies combined qualitative rating scales with single overall quantitative scores in 10-point and 13-letter scales. Additionally, the WFPS resulted in scores under a 12-point scale. This diverse range of rating scales highlights the variability in assessment approaches and underscores the need for standardization to ensure consistent and reliable evaluations across different studies.

### 3.4 Critical analysis of performance assessment methods

Our review primarily focuses on the methods of performance assessment methods. In this sense, for all studies, the primary outcome of all publications comprised the development, validation, and/or implementation of a music performance assessment. Nonetheless, we present below some of the most relevant complementary findings across studies. Table 3 synthesizes the objectives and findings for each study.

**Table 3:** Objectives and synthesized findings of included studies.

Author (year)	Objectives	Findings
Abeles (1973)	To examine a technique for the development of performance rating scales to measure achievement.	The three major results of the study were: (1) a 30-item rating scale based on a six-factor structure of clarinet music performance; (2) high inter-judge reliability estimates for both the total score (> 0.9) and the scale scores (> 0.6); and (3) criterion-related validity coefficients >0.8. Such results suggest that the facet-factorial approach can be an effective technique for the construction of rating scales to measure complex behavior such as music performance.
Fiske (1975)	To examine differences in trumpet performance assessment between brass and non-brass judges, and wind and nonwind judges.	The results showed no significant differences between brass and non-brass judges. Technique was found to be rated significantly different when wind judges were compared with nonwind judges, and it was also the most distinct trait when the groups of judges were combined into a single judge group and the five rated traits were intercorrelated.
Mills (1987)	To analyze the assessment of solo musical performance in the Western Classical tradition and to offer a model which can be used to better measure solo music performance.	The results showed that a high proportion of the variance in the overall marks can be accounted for by linear and quadratic equations in the bipolar “constructs,” meaning that the overall marks can be explained in terms of characteristics which can be understood by nonspecialists, and which are not related to instrument-specific technique. The proportion of variance in rank accounted for was hardly less among nonspecialists than among music specialists; overall marks given by nonspecialists seem as “rational” as those given by specialists.
Zdzinski (1991)	To review studies dealing with solo instrumental music performance measurement and their implications for future research in performance measurement.	The Watkins-Farnum Performance Scale (1954) has been widely used in music education research as a measure of performance achievement despite its seemingly validity problems. Other studies have attempted to improve musical performance evaluation by replacing ratings based on overall impressions with more systematic rating scales and by using evaluative criteria that sample performance areas. Reliability and validity data for these studies seem promising (Abeles, 1973; Bergee, 1987), suggesting that common judging criteria help to improve musical performance evaluation. Another promising area regards the advances in acoustic and computer-assisted measurement, suggesting that several performance parameters can be judged with great accuracy and increased reliability.
Bergee (1993)	To explore the efficacy of peer and self-evaluation of applied brass jury performances considering faculty evaluation as standard of comparison. Second, to assess the effects of videotape vs. live performance and internal vs. external adjudicators on evaluation.	Inter-judge reliability for faculty and peer evaluation panels generally was high, with total score correlations ranging from 0.83 to 0.89 (*p* < 0.01). Correlations among faculty and peer-group evaluations also were high, with total score *r* ranging from 0.86 to 0.91 (*p* < 0.01). Data indicated consistent agreement on factors describing musical effectiveness, tone quality/intonation, and technique. Rhythm-tempo, however, revealed less consistency of agreement. Consonant with prior investigations, self-evaluation correlated poorly with faculty and peer evaluation. The effects of videotaped performances were minimal and prior knowledge of performers did not seem to affect evaluations.
Winter (1993)	To examine the effects of training and experience on qualified musicians’ and music educators’ judgments.	The results suggested that the training a music examiner receives prior to the performance assessment session may be more important in producing consistent and accurate reports than the amount of previous examining experience. The criteria used by the music examiner should be clearly presented with appropriate dimensions for the musical instrument on which the student performs.
Saunders and Holahan (1997)	To determine the suitability of the use of criteria-specific rating scales in the selection of high school students for participation in an honors ensemble.	The rating scales yielded substantial variability and moderately high-to-high alpha reliabilities. Different judges collectively demonstrated a consistency of performance evaluation results. The data presented provided indirect evidence that criteria-specific rating scales have superior diagnostic validity than Likert-type rating scales and traditional open-ended rating forms. Stepwise multiple regression indicated that student total scores could be predicted from scores of five major dimensions: tone, technique/articulation, rhythmic accuracy, interpretation, and sight-reading-interpretation (Multiple *R* = 0.96).
Davidson and Coimbra (2001)	To examine issues related to assessing biases and development of assessment criteria by studying the case-study of the evaluation processes undertaken by a panel assessors of mid-term recitals at the Guildhall School of Music.	The major categories assessors relied on for their evaluation were: body communication, technique, and presentation of musical content (i.e., emotional expression, personality of the interpreter). The assessors showed a high degree of correlation in their assessment grades and the way in which they discussed individuals, indicating that they shared similar ideas. Nonetheless, the criteria for the assessments were implicit rather than explicit: in one hand, results suggested that there was a shared code of assessment criteria between assessors; on the other, the lack of articulated criteria means that no individual assessor is certain of what beliefs (personal or others’) drove decisions.
Stanley et al. (2002)	To investigate examiner perceptions of the effects of introducing criteria into music performance assessment procedures at a tertiary conservatorium of music.	In discussing their music performance assessment strategies examiners described holistic and criteria-specific approaches. Some examiners felt using criteria helped them focus on important assessment issues and that criteria were useful for articulating desirable performance characteristics in feedback to students. Other examiners believed criteria-based assessment represented a narrow view which tended to interfere with their holistic assessments of music performance. Discussions generated a new assessment system to be implemented in this pedagogical context.
Zdzinski and Barnes (2002)	To develop a valid and reliable assessment measure for stringed instrument performance.	The factor analysis of an initial pool comprising 90 assessment items resulted in the detection of five principal factors (interpretation/musical effect, articulation/tone, intonation, rhythm/tempo, and vibrato) and the selection of 28 items for the subscales of the SPRS. Reliability varied from 0.873 to 0.936 for each judging panel. Two studies were conducted to establish criterion related validity, with correlations ranging from 0.605 to 0.766 between the SPRS and two other rating scales.
Bergee (2003)	To examine the inter-judge reliability of faculty evaluation of end-of-semester applied music performances considering the variables variability in size of adjudication groups, mode of evaluation, and adjudication experience.	Full-panel inter-judge reliability was consistently good regardless of panel size. All total score reliability coefficients were statistically significant, as were all coefficients for the global letter-grade assessment. All subscale reliabilities for all groups except Percussion (which, with an *n* = 2, had a stringent significance criterion) were statistically significant, except for the Suitability subscale in Voice. For larger panels (*n* = of 4 and 5), rating scale total score reliability was consistently but not greatly higher than reliability for the letter-grade assessment. There was no decrease of average reliability as group size incrementally decreased. Permutations of two and three evaluators, however, tended on average to exhibit more variability, greater range, and less uniformity than did groups of four and five. No differences in reliability were noted among levels of experience or between teaching assistants and faculty members. Use of a minimum of five adjudicators for performance evaluation in this context was recommended.
Thompson and Williamon (2003)	To develop a research tool by examining some of the assumptions and implications inherent in any formal system of musical performance assessment, and to illustrate some of these by reporting data from an empirical study.	Correlations between evaluators were moderate and some evidence of bias according to the evaluators’ own instrumental experience was found. The use of a larger n of evaluators is recommended to fade away individual differences. Strong positive correlations were found between items on the assessment scheme, indicating an extremely limited range of discrimination between categories. This can be attributed to semantic problems which can be solved by providing more precise guidelines, defining each category in detail.
Barry (2009)	To explore some of the key topics related to music performance evaluation including significant political and social issues, pitfalls and concerns.	While both formal and informal evaluations are inherent and essential aspects of music learning and performance, the particulars of how to carry out evaluation as well as how the results of evaluation should be used remain controversial. Apart from presenting the example performance evaluation tools, the author also provides instructions on how to develop such instruments.
Ciorba and Smith (2009)	To investigate the effectiveness of a multidimensional assessment rubric administered to all students performing instrumental and vocal juries at a private Midwestern university during one semester.	Inter-judge reliability coefficients indicated a moderate-to-high level of agreement among judges. Internal reliabilities were consistent within each performing area. Results also revealed that performance achievement was positively related to participants’ year in school, which indicates that a multidimensional assessment rubric can effectively measure students’ achievement in solo music performance. High correlations among scale dimensions were found. Although the unique contribution of each score to the composite may be limited, the comparison of scores in different dimensions presents a profile of student achievement that can lead to plans for future instruction to address areas of weakness, supporting the benefits of criteria-based systems in comparison to overall ratings.
Russell (2010a)	To identify the underlying aural factors of guitar performance by developing a guitar performance rating scale using facet-factorial techniques.	The results of a factor analysis applied to an initial pool of 99 item statements yielded a five-factor structure comprising interpretation, tone, technique, rhythm/tempo, and intonation. These factors accounted for approximately 71% of the total variance. The selection of the 32 items chosen to represent the factors of the Guitar Performance Rating Scale (GPRS) was based on factor loadings. Alpha reliability for the GPRS was estimated at 0.962 for the 32-item scale.
Wrigley and Emmerson (2013)	To investigate ways to improve the quality of music performance evaluation in tertiary music education.	Findings suggested that, although several construct and general dimension commonalities were found among the items across all scales, the presence of significant instrument-specific differences indicated that the use of generic rating scales may not provide sufficient content validity. This study demonstrated that disciplinary objectivity in music performance assessment could be empirically defined and measured within an ecologically valid framework at a tertiary-level Australian music institution using a rigorous combination of qualitative and quantitative methodologies. Each of the PERS models (piano, voice, strings, brass, woodwind) provided acceptable levels of reliability and construct validity. High internal reliabilities were found with each of the PERS factors, with alphas ranging from 0.81 to 0.98.
Russell (2015)	To test a hypothesized model of solo music performance assessment, considering the influence of technique and musical expression on perceptions of overall quality.	The analysis of the performance data in relation to the proposed model demonstrates a significant and positive causal relationship between technique and musical expression. Results indicated the ability to predict increases in the perception of overall quality both directly and indirectly through technique and musical expression. Technique demonstrated direct effects on overall quality and expression, while expression demonstrated direct effects on overall quality only. Results suggest that deficiencies in technique will not only influence assessments of technique, but also musical expression and overall perception of performance quality.
Costa and Barbosa (2015)	To contrast the assessments done by trumpet’s teachers, based on Scale of Evaluation of the Musical Execution, with the free assessments carried out by the same group of teachers.	By comparing the two forms of evaluation, we verify the inconsistency of the assessments and judgments in respect to the performance of the students. Although results showed high inter-judge variability in both evaluation models, variability increased in evaluations without pre-defined criteria. Additionally, our results show that Trumpet’s teachers´ evaluation of the students´ instrumental performance is mostly focused on two dimensions: materials and expression, which are stages at the most basic levels of the Spiral Theory.
Wesolowski et al. (2017)	To describe the development of a valid and reliable rubric to assess secondary-level solo instrumental music performance based on principles of invariant measurement.	The result was the development of the Music Performance Rubric for Secondary-Level Instrumental Solos (MPR-2L-INSTSOLO), a 30-item rubric consisting of rating scale categories ranging from two to four performance criteria. The scale displayed overall good psychometric qualities (reliability, precision, and validity). This is the first music performance assessment measure developed using item response theory techniques and, more specifically, Rasch measurement techniques.
Álvarez-Díaz et al. (2021)	To design and validate an analytical evaluation rubric allowing for the most objective evaluation possible of a musical solo performance in a regulated official competition.	The essential unidimensionality of the rubric was confirmed. The results of the PCA indicated that the five criteria can be summarized in a single factor accounting for 80% of the variance. No differential effects between raters were found, nor were significant differences seen in each rater’s internal consistency.

Five studies (and seven assessment systems) (Abeles, 1973; Bergee, 1993, 2003; Jones, 1986; Nichols, 1991, as cited in Bergee, 2003; Russell, 2010a; Zdzinski and Barnes, 2002) used facet-factorial approaches, defined as conceptualizing the behavior as multidimensional and selecting scale items through factor analysis, validating the method as an effective technique for the construction of rating scales. These studies collected a pool of initial items (range: 90–99) generated by experts, to which factorial techniques were applied to produce a final version of the measurement instrument that included items with high factor loadings (range: 27–32). Zdzinski and Barnes (2002) found that the factor grouping slightly differed from those in Abeles (1973) and Bergee (1993), most likely due to instrument-specific technical requirements. For example, for strings, tone and articulation were grouped in the same factor (Zdzinski and Barnes, 2002); for brass, tone and intonation were grouped and technique was accommodated in another factor (Bergee, 1993); and for woodwinds, separate factors were established for tone, articulation, and intonation (Abeles, 1973). The SPRS was the only system that included vibrato items in a separate factor. Similarly, the Jones Scale (Jones, 1986) yielded a different factor structure with Interpretation/Musical Effect as common and other factors consisting of Tone/Musicianship, Technique, Suitability/Ensemble, and Diction. The piano scale developed by Bergee (2003) consisted of only three factors (Interpretation/Musical Effect, Rhythm/Tempo, and Technique).

Wrigley and Emmerson (2013) developed PERS models for five instrument families (piano, voice, strings, brass, woodwind) distilling acceptable levels of reliability (internal reliability alphas ranging from 0.81 to 0.98) and construct validity. Their results also confirmed the importance of using instrument-specific scales, as, although the authors found consistency between instrument departments at the general factor of evaluation, they also found considerable variation between dimension constructs, which can be attributable to instrumental idiosyncrasies. Moreover, this was the only work identified in which the same author team developed evaluation systems for five instrumental families. Wesolowski et al. (2017) recently applied the Multifaceted Rasch Partial Credit Measurement Model to create a 30-item solo wind performance assessment rubric. In summary, Rasch techniques enable construct-irrelevant factors, such as individual characteristics of persons, raters, or items, to not interfere between observed data and predictions of the model, accounting for multiple issues related to individual variability observed in facet-factorial approaches. The scale displayed overall good psychometric qualities (reliability, precision, and validity).

Regarding assessment systems transversal to multiple instruments, Mills (1987) found that a bipolar scale effectively explained a high proportion of variance in overall ratings. Ciorba and Smith (2009) developed a multidimensional assessment rubric, applicable across instruments and university years, that revealed moderate to high levels of agreement among judges and was influential in measuring students’ achievement, as proved by the positive correlation between performance achievement and participants’ year in university (freshman, sophomore, junior, and senior). Recently, Álvarez-Díaz et al. (2021) also validated a unidimensional assessment rubric applicable across instruments.

Russell (2015) introduced novel findings regarding the weight of each performance dimension, demonstrating a positive causal relationship between technique and musical expression. Technique showed direct effects on the ratings of overall quality and musical expression, while musical expression demonstrated direct effects on overall quality only, suggesting that deficiencies in technique will not only influence assessments of it but also of musical expression and the overall perception of performance quality.

In a literature review, Zdzinski (1991) discussed that despite the widespread application of the Watkins-Farnum Performance Scale (1954) in music performance research up to date, studies have shown moderate and low validity coefficients (e.g., 0.63, 0.40, 0.12) when comparing the WFPS with other scales. Moreover, the WFPS is based on calculating a score derived from bar-by-bar performance errors (e.g., rhythm, pitch), which poses multiple drawbacks: (a) the final score does not allow for differentiation of errors as they are summed; (b) only one point (corresponding to one error) can be deducted by measure regardless of the number of errors occurring; and (c) the score does not include parameters such as musicality, phrasing, or intonation. The author also highlighted that systematic and criteria-based assessment systems such as the ones by Abeles (1973) or Bergee (1993), yielded promising results in terms of reliability and validity. Saunders and Holahan (1997) and Barry (2009) also emphasize that, although more challenging to build, criteria-specific rating scales have superior diagnostic validity than Likert-type rating scales and traditional open-ended rating forms. In line with these findings, Costa and Barbosa (2015) discovered that variability within trumpet judges increased in evaluations without pre-defined criteria, although it was generally high in both free and criteria-based evaluation models. In fact, multiple studies reported high correlations between performance assessment categories (Álvarez-Díaz et al., 2021; Ciorba and Smith, 2009; Fiske, 1975; Thompson and Williamon, 2003), underscoring that, although the unique contribution of each score to the composite may be limited, the comparison of scores in different dimensions presents a profile of student achievement that can be transposed into valuable feedback related to specific performative skills and lead to plans for future instruction to address areas of weakness.

From a complementary perspective, Davidson and Coimbra (2001) found that, although assessors demonstrated high degrees of correlation in grades, their criteria were implicit rather than explicit. Assessors seemed to share a code of assessment criteria but lacked articulation and delimitation, suggesting that they were uncertain of what their own or others’ beliefs drove decisions. In the interview study by Stanley et al. (2002), examiners at a tertiary music conservatorium presented mixed opinions regarding criteria-based assessments. While some felt using criteria facilitated the focus on essential assessment issues and was helpful in articulating desired performance characteristics in feedback to students, others believed criteria-based assessment represented a narrow view that tended to interfere with their holistic assessments of music performance. Discussions with examiners led to the adaptation of the conservatorium’s assessment system, considering their preference for fewer criteria so that more time could be dedicated to writing detailed comments (Stanley et al., 2002).

Regarding mediator factors in performance assessment, studies reported no differences between brass and non-brass judges (Fiske, 1975), nor between music specialists and nonspecialists (Mills, 1987). Nevertheless, in Mills (1987), the constructs used did not require possessing musical knowledge (e.g., the performance was hesitant/fluent). Fiske (1975) also found that technique was rated differently between wind and nonwind judges (Fiske, 1975), and Thompson and Williamon (2003) reported evidence of bias according to examiners’ instrumental expertise. Bergee (1993) found high inter-judge reliability for faculty and peer evaluation panels, demonstrating consistent agreement on all factors but rhythm-tempo; self-evaluation, however, correlated poorly with faculty and peer evaluation. No differences were found between levels of evaluative experience or between teaching assistants and faculty members (Bergee, 2003). In fact, Winter (1993) found that the prior training received by music adjudicators was more significant in producing consistent and accurate assessments than the previous experience in such a role. Finally, Bergee (2003) found that inter-judge reliability was consistently good regardless of panel size, although permutations of two and three evaluators tended to exhibit more variability, greater range, and less uniformity than did groups of four and five. Hence, the author recommended using at least five adjudicators for performance evaluation. Furthermore, the same study found no effects of videotaped (versus live) performances or prior knowledge of performers.

## 4 Discussion

### 4.1 Main findings

This systematic review summarized solo music performance assessment methods reported in published scientific research for over 50 years. Significant heterogeneity was observed between the included studies regarding the assessment systems used to evaluate performances, allowing for the retrieval and analysis of 26 different systems reported across 20 publications. We found 11 generalized, six family-, and nine instrument-specific scales, among the identified systems. Some studies advocate for adopting family- and instrument-specific scales that consider the idiosyncrasies related to instrumental and vocal technique. For example, in assessing vocal performance, diction and language facility are relevant skills (Jones, 1986), just as breathing, air support, and tongue are for winds (Bergee, 1993; Wesolowski et al., 2017) or vibrato for strings (Zdzinski and Barnes, 2002). The argument for instrumental scales is further supported by findings such as rating differences between wind and nonwind judges in the technical dimension (Fiske, 1975), evidence of bias according to examiners’ instrumental expertise (Thompson and Williamon, 2003), substantial variability between instrument departments on the level of dimension constructs (Wrigley and Emmerson, 2013), and factor grouping of assessment items varying between instrumental families [e.g., the String Performance Rating Scale by Zdzinski and Barnes, 2002 yielded a different factorial organization than the Clarinet Performance Rating Scale by Abeles, 1973]. On the contrary, generalized scales seem to facilitate the standardization of assessment practices across instrumental and vocal departments and foster the development of a common criteria vocabulary among examiners, a previously identified deficiency (Davidson and Coimbra, 2001). After carefully considering examiners’ opinions, requesting fewer criteria and more space for subjective comments, one tertiary music conservatorium replaced a family-directed assessment system with a set of common assessment criteria (Stanley et al., 2002). Generalized systems have been successful in contexts where direct comparisons are desired, for instance, in measuring students’ achievement throughout university years (Ciorba and Smith, 2009), in multi-instrument competitions (Álvarez-Díaz et al., 2021), or in music performance assessment by non-experts (Mills, 1987). We conclude that, as posed by Barry (2009), there is no “one-size-fits-all approach to music evaluation” and that, depending on the context, function of the assessment, and institutional culture, both generalized and instrument-oriented methods can be effectively implemented.

Regarding the main assessment categories, most assessment systems adopted a structure comprising one technical category, one interpretative/expressive category, and multiple musical feature categories (e.g., pitch, rhythm, intonation). Additionally, eight systems reserved one category for stage presence, and even fewer encompassed categories for aesthetics and epoque adequacy, and body behavior. Although this structure seems reasonable, one may reflect on how technique relates to both musical effect execution and interpretation. Musical execution and communication are only attainable if the performer possesses substantial skill levels in their instrument, supporting the priority to developing a precise technique in music education settings (Gellrich and Parncutt, 1998; McPherson, 2022). For example, clarinet players’ finger movements in pressing and releasing keys, together with breathing, determine the timing of tone onsets (also known as tempo or rhythmic accuracy in the categories of assessment systems) (Palmer et al., 2009). Similarly, violin players need to master upper body movements to express melodic continuity through timing (*rubato*), a common marker of personal interpretation (Huberth et al., 2020). Russell (2015) findings further support this notion, showing that technique directly impacts the ratings of overall quality and musical expression, while musical expression solely impacts the overall quality. Hence, technical deficiencies affect not only on technique ratings but also on the perception of musical expression and the overall performance quality. In accordance, Álvarez-Díaz et al. (2021) attributed the higher assessment weights (30% each) to the technical level and performance quality, followed by the difficulty of chosen pieces (20%), stylistic coherence, and stage presence (10% each). This categorical intertwinement has also been noted through inter-category correlations in several studies (Álvarez-Díaz et al., 2021; Ciorba and Smith, 2009; Fiske, 1975; Thompson and Williamon, 2003). Considering these findings, we believe it is worth reflecting on the weight given to the technical category in relation to others and to what extent it could be pertinent to aggregate skills related to musical features, such as pitch, intonation, or articulation, in this sector.

By analyzing the rating scales implemented, we identified that most assessment systems used 5-point Likert qualitative scales, which reflect the evaluators’ level of agreement with a set of assessment elements. However, we noted that, gradually, more recent studies started replacing these with rubrics, which provide detailed descriptions for each level of the achievement scale. Such descriptions constitute beneficial feedback for the evaluated individuals, as they present a clearly defined set of descriptors related to learning expectations, providing both a measure of the present performance and information to improve future performances (Ciorba and Smith, 2009). Moreover, rubric descriptors also facilitate the examiners’ role by delimiting the expected outcomes for each level, again promoting the much-needed common understanding of assessment criteria (Wesolowski, 2012). In terms of the number of levels within scales, consensus among authors appeared challenging to reach. Most kept to traditional 5-point Likert scales (e.g., Bergee, 2003; Saunders and Holahan, 1997), while some selected even numbered scales (e.g., 4-point) to eliminate neutral categories by forcing positively or negatively positioned responses (Mills, 1987; Wesolowski et al., 2017), and others adopted 1–10 quantitative scales due to their direct relation with the 100-point scale frequently used in music educational contexts (Thompson and Williamon, 2003). Research has shown that scales with more than 10 points result in decreased reliability, although they provide respondents with increased precision levels (Preston and Colman, 2000), and that 5-point scales can produce inconsistent answer scores (Toepoel and Funke, 2018). Curiously, seven-point scales seem to be the best compromise (Krosnick and Fabrigar, 1997; Maitland, 2009), and they were only adopted in the PERS (Wrigley and Emmerson, 2013). Nevertheless, it is crucial to highlight that, in developing rubrics, implementing a high number of levels can be challenging, as it becomes more difficult to define differences between expected outcome descriptors.

One surprising finding regards the almost imperceivable differences in assessment criteria between diverging types of populations. Most of the studies focused on either high schoolers or university students, representing distinct performance levels. Hence, we expected that, at the item level, descriptions would be adapted to the expected skill competence for each learning stage. However, the descriptions were general to the extent to which most items were applicable to multiple populations. For example, when presented with the following item: command of instrument is (select one option) below minimum expectations/demonstrates potential for musical growth/has achieved a point where musical maturity is possible/is proficient/demonstrates potential for professional success (Ciorba and Smith, 2009), judges are unable to infer what is, indeed, the expected command of instrument for the student at hand. For example, for a beginner saxophone player, producing sound without squeaking would be a good demonstration of the command of the instrument, while for a superior-level student, it could be the ability to play harmonics while maintaining intonation and timbre quality. Barry (Barry, 2009) introduces a fine example of a rubric adapted for a preparatory piano exam in which descriptors are objective and level-appropriate (e.g., not yet – student misses standard fingering more than once; almost – student misses standard fingerings once; … exceeds standard – student develops more efficient fingering practice). Without a doubt, music performance assessment, unlike more objective disciplines, is particularly defying due to the involvement of expressive decisions and response divergence (Wesolowski, 2012). Moreover, it has been discussed that music educators, in particular, face challenges in systematically documenting and quantifying the essential concepts and skills they want their students to acquire and demonstrate at different levels of performance achievement (Payne et al., 2019; Wesolowski, 2015). Therefore, we postulate that the level of accuracy the assessment systems lacked in determining the specific goals for each learning stage may be a reflection of the path music education has yet to pursue to reform outdated practices and adopt more effective, efficient, and clearly defined methods for measuring student growth, aligning with other general education policies.

### 4.2 Limitations

Two main limitations were identified in this work. First, we included only reviews and original research published in peer-reviewed journals. Citation searches revealed that numerous studies on music performance assessment exist in grey literature, such as doctoral dissertations and institutional pedagogical guidelines. However, many assessment systems initially presented in these were later converted into articles by the same authors or implemented by others. Therefore, we focused this review on published, peer-reviewed works to ensure validity and scientific rigor, even though it may have implicated discarding additional publications. Second, our review’s scope limits our ability to draw conclusions about the efficacy of the assessment systems reported. We focused on their construction, characterization, and validation rather than analyzing replication studies. While the assessment systems analyzed generally reported good reliability and consistency in their original studies, subsequent research might have identified weaknesses. For instance, Zdzinski (1991) noted in his literature review that multiple *post hoc* studies using the Watkins-Farnum Performance Scale (1954) had already revealed moderate and low validity coefficients compared to other scales. Future research should map the use of various assessment systems post-implementation, providing insights into their frequency of use and into additional validity results.

## 5 Conclusion

In conclusion, this review documents the major progress in music performance assessment simultaneously underscores the imperative for continued research to address persistent gaps and improve existing methodologies. We investigated music performance assessment systems found in scientific literature, analyzing their corresponding evaluation categories and descriptive criteria, rating methodology, and target audience. A total of 51 full-text publications were assessed for eligibility, which were reduced to 20 articles that met the inclusion criteria.

The literature review identified 26 assessment systems for detailed analysis. Most studies evaluated high school and university students, with evaluators primarily being music teachers and faculty members. About one-third of the studies assessed a heterogeneous group of instrumental and vocal performances, while the others focused on specific instruments/voice or instrumental families. Consequently, most assessment systems were designed for use across various instruments, though some were family- or instrument-specific. Many systems followed a structural logic including one technical category, one interpretative/expressive category, and multiple musical feature categories (e.g., pitch, rhythm, intonation), further expanded into descriptive items. Five-point Likert qualitative scales were most common, though recent studies showed a trend towards rubrics for detailed feedback, facilitating both examiners comprehension and student progress. Interestingly, no differences were found in assessment criteria for students at different learning stages. Research efforts should be directed toward developing and validating assessment criteria specific to different proficiency stages. Customizing assessment tools to meet the needs of beginners, intermediate, and advanced students is crucial. It allows educators, researchers, and curriculum developers to offer more relevant and constructive feedback, a contribution that is crucial for fostering individual growth and progress in music performance. Also, this strategy ensures that assessment methods are suitably challenging and developmentally appropriate for each level of a student’s educational journey.

By delimiting and characterizing the existing assessment systems, this study represents a novel contribution for educators and policymakers looking to enhance curriculum design and instructional practices in music education, as well as for researchers aiming to design science-based, objective performance assessment studies. With continued efforts in these areas, we can look forward to a future where music performance assessments are more reliable, equitable, and truly support and enhance the musical journey of every student.

## Data Availability

The original contributions presented in the study are included in the article/supplementary material, further inquiries can be directed to the corresponding author/s.
